# Selenium-Cultured *Potamogeton maackianus* in the Diet Can Alleviate Oxidative Stress and Immune Suppression in Chinese Mitten Crab (*Eriocheir sinensis*) Under Copper Exposure

**DOI:** 10.3389/fphys.2020.00713

**Published:** 2020-06-19

**Authors:** Jun Qiang, Xue-Jun Duan, Chuan-Kun Zhu, Jie He, Jin-Wen Bao, Yi-Fan Tao, Hao-Jun Zhu, Pao Xu

**Affiliations:** ^1^Key Laboratory of Freshwater Fisheries and Germplasm Resources Utilization, Ministry of Agriculture, Freshwater Fisheries Research Center, Chinese Academy of Fishery Sciences, Wuxi, China; ^2^Adult Education College, Wuxi Institute of Technology, Wuxi, China; ^3^Jiangsu Collaborative Innovation Center of Regional Modern Agriculture & Environmental Protection, Huaiyin Normal University, Huai’an, China

**Keywords:** selenium, Chinese mitten crab (*Eriocheir sinensis*), *Potamogeton maackianus*, oxidative stress, immune regulation, Cu^2+^ exposure

## Abstract

Selenium (Se) is an essential trace element for aquatic animals. The aquatic plant *Potamogeton maackianus* is an important natural food of Chinese mitten crab (*Eriocheir sinensis*). The aim of this study was to determine whether the antioxidant and immune responses of Chinese mitten crab are affected by including Se-cultured *P. maackianus* in the diet. Three groups of *P. maackianus* were cultured at levels of 0.02 mg/kg Se, 8.83 mg/kg Se, and 16.92 mg/kg Se, and the plants in these groups were used in experimental diets fed to crabs (dietary Se content of 0.05, 0.43, and 0.82 mg/kg, respectively). Compared with crabs in the 0.05 mg/kg group, those in the 0.82 mg/kg group showed significantly increased specific growth rate, protease and lipase activities, triglyceride and cholesterol contents, and Se content in the hepatopancreas and muscle (*P* < 0.05); increased activities of glutathione peroxidase, glutathione reductase, and catalase in the antioxidant system; increased transcript levels of *MT* (encoding metallothionein); and decreased malondialdehyde content (*P* < 0.05). At the end of the rearing experiment, the crabs in the different groups were exposed to copper (Cu^2+^) stress for 96 h. All the juvenile crabs in the 0.43 and 0.82 mg/kg groups survived 96 h of Cu^2+^ stress. Crabs in the 0.82 mg/kg group showed enhanced antioxidant responses under Cu^2+^ stress, increased transcript levels of *MT* and *LYZ*, and increased resistance. Therefore, supplementation of the diet of Chinese mitten crab with increased levels of Se-cultured *P. maackianus* can reduce oxidative stress under Cu^2+^ exposure, activate the immune response, and benefit growth.

## Introduction

Selenium (Se) is present in all tissues and cells of the body and is an essential trace element for animals (Watanabe et al., 1997). It participates in regulating various physiological processes and exerts various biological functions in the form of selenoproteins (Lu et al., 2012; Nugroho and Fotedar, 2013). As the biological activity of Se and its mechanism of absorption and metabolism in the body are becoming better understood, the use of Se as a dietary supplement for aquatic animals is becoming more common (Schrauzer, 2003; Neamat-Allaha et al., 2019). The glutathione peroxidase (GSH-Px) family is an important group of selenoproteins. The main function of its members is to promote hydroperoxide metabolism and reduce oxidative damage in the body (Schrauzer, 2003). Dietary supplementation with Se has been shown to affect the growth performance, antioxidant capacity, and immune regulation of crustaceans. Wang et al. (2019) found that dietary supplementation with Se-yeast significantly improved superoxide dismutase (SOD), GSH-Px, and acid phosphatase (ACP) in Chinese mitten crab (*Eriocheir sinensis*, commonly known as river crab), and enhanced its ability to resist nitrite stress. Qin et al. (2016) found that dietary supplementation with 0.2 g/kg nano-Se promoted the growth and health of Chinese mitten crab and increased antioxidant capacity after hypoxia stress. Chiu et al. (2010) found that dietary supplementation with Se significantly improved phenol oxidase and phagocytosis activities and the antioxidant capacity of giant freshwater prawn (*Macrobrachium rosenbergii*), and enhanced its ability to resist *Debaryomyces hansenii* infection. However, a low level of Se in the diet can lead to a series of oxidative stresses in oriental river prawn *Macrobrachium nipponense* (Kong et al., 2017).

Se can be supplied in the diet in three forms: inorganic (e.g., sodium selenite) (Kouba et al., 2014), organic (e.g., Se-yeast and Se-methionine) (Thomas and Janz, 2011; Wang et al., 2019), and nano-Se (Neamat-Allaha et al., 2019). Inorganic Se has low utilization, high toxicity, and environmental risks, because its residue can pollute the environment (Kouba et al., 2014). The advantages of Se-yeast and Se-methionine as dietary supplements are their high efficiency and low environmental risks, but high concentrations may cause physiological stress (Thomas and Janz, 2011). The biological activity of nano-Se is higher than that of inorganic Se and organic Se, but its disadvantages include a complicated preparation process and high cost (Neamat-Allaha et al., 2019). For these reasons, they are rarely used as additives in aquatic feed. In a recent study common barbel (*Barbus barbus*) fed dietary Se-enriched microalgae biomass showed significantly increased Se contents in muscle and liver. The activities of glutathione reductase (GR) and catalase (CAT) were increased while being less toxic (Kouba et al., 2014). Yuan et al. (2018) found Chinese mitten crab fed Se-biofortified corn increased in total muscle Se levels and activities of alkaline phosphatase (ALP), lysozyme (LYZ), GSH-Px, and SOD. In addition, as a functional food added to the diet, Se-cultured garlic fed to African catfish (*Clarias gariepinus*) increased growth and Se concentrations in fish filets (Schram et al., 2008, 2010). It is possible to convert inorganic Se into organic Se through feeding aquatic animals on Se-cultured aquatic plants or bait (Mechora et al., 2013).

Chinese mitten crab has delicious meat containing abundant unsaturated fatty acids. It is one of the main farmed crustaceans in China. Recently, the role of aquatic plants in the cultivation of mitten crab has received increasing attention. Aquatic plants are not only rich in nutrients, they are a natural bait and an important source of nutrition for mitten crabs, and can provide habitat and shelter for crabs during molting (Li, 2010). Aquatic plants can also purify the water, thereby improving crab production and growth quality (Wen et al., 2012). *Potamogeton maackianus* is a common dominant species in submerged vegetation in grass-type lakes in the middle and lower reaches of the River. This species plays an important role in freshwater ecosystems and lake fisheries. It is also a natural biological bait for Chinese mitten crab and is rich in nutrients (Zhang et al., 2008). Therefore, the first aim of this study was to determine whether dietary supplementation with Se-cultured *P. maackianus* can increase the Se content in mitten crab and enhance its antioxidant capacity.

With the continuous expansion of Chinese mitten crab farming, pollution of aquaculture water is also increasing. Water bodies are affected by industrial, agricultural, and domestic sewage. In addition, aquaculture water is often polluted by large amounts of copper sulfate (Sroda and Cossu-Leguille, 2011), which is used for disease control and to clear algae (De Romaña et al., 2011). Although Cu^2+^ is an essential nutrient for aquatic animals, excessive Cu^2+^ in the aquatic environment or in the diet can negatively affect crustacean immunity, leading to tissue oxidative damage and liver necrosis that increase susceptibility to pathogens and cause disease outbreaks (Qiang et al., 2019). Nutrients have complex mutual promoting or inhibitory effects in the body, and Se has been shown to increase antioxidant defenses (Trevisan et al., 2011) and alleviate symptoms of poisoning and apoptosis caused by heavy metals in animals (Wang et al., 2013; Xu et al., 2016). However, whether Se has the same function in resisting heavy metal stress in aquatic organisms is unknown. Therefore, the second aim of this study was to determine whether Chinese mitten crab fed increasing Se content in their diet showed enhanced antioxidant capacity and reduced oxidative damage under heavy metal stress. The results of this study provide information about the slow-release effect of Se, and a reference for the sustainable development of aquaculture and control of the quality and safety of aquatic products.

## Materials and Methods

### Cultivation of *P. maackianus* in Different Concentrations of Se

*Potamogeton maackianus* was collected from the Taihu Lake Basin in Jiangsu Province, China. Plants growing to a basically uniform height of 15–20 cm were selected and transplanted into a mud substrate in a laboratory culture tank. Ten strains per culture tank and three replicates per experimental concentration were set up. The experiment used tap water, and chemically pure Na_2_SeO_3_ was added to adjust Se in the water medium to three concentrations: 0, 20.94, and 67.68 mg/L. Before the experiment, 1 mg/mL Na_2_SeO_3_ mother liquor was prepared. During the experiment, the light intensity in the water column of the cultivation tank was 110–240 μE/m^2^/s, the water pH was 6.9–7.4, the water temperature was 25–29°C, and the water was changed once a day. After cultivation for 12 days, the plant stems and leaves were collected, washed, shredded, placed in a cold air dryer at −40°C, and dried for 48 h. We used a pulverizer to powder the dried material, then passed it through a 40-mesh sieve, and stored it in a refrigerator at 4°C for future use. The nutritional composition of each *P. maackianus* group is shown in the Supplementary Tables S1, S2. Inductively coupled plasma mass spectrometry (ICP-MS, Agilent 7700, United States) was used to determine the Se content in the *P. maackianus* powder, and this was 0.02, 8.83, and 16.92 mg/kg.

### Experimental Design and Preparation of Diets

The feed formulae and nutritional composition are shown in Table 1. Casein was the main protein source and fish oil was the main fat source. The three *P. maackianus* powders with different Se contents were added to the basic feed to prepare three groups of isonitrogenous and isolipidic experimental diets (crude protein 40.17%, crude fat 9.74%). When preparing the feed, all diet ingredients were passed through an 80-mesh sieve, weighed, and mixed according to the specified proportions, then fish oil and an appropriate amount of water were added. The mixture was processed by a screw extruder into particles with a diameter of 1 mm. The particles were dried at 4°C and then stored at −20°C.

**TABLE 1:** Ingredients and proximate composition of experimental diets.

	**Group 1**	**Group 2**	**Group 3**
**Ingredients**			
Casein^1^	46	46	46
Corn starch	26.5	26.5	26.5
Fish oil^2^	8	8	8
Soybean lecithin oil^3^	2	2	2
Vitamin premix^4^	1	1	1
Mineral premix^5^	2	2	2
Choline chloride	0.5	0.5	0.5
Vitamin C phosphate	0.1	0.1	0.1
NaCl	0.4	0.4	0.4
Ca(H_2_PO_4_)_2_	2.0	2.0	2.0
Betaine	0.5	0.5	0.5
Carboxymethyl cellulose	3.0	3.0	3.0
Microcrystalline cellulose	3.0	3.0	3.0
0.02 mg/Kg Se in *P. maackianus*	5.0		
8.83 mg/Kg Se in *P. maackianus*		5.0	
16.92 mg/Kg Se in *P. maackianus*			5.0
Total	100.0	100.0	100.0
**Nutrient composition**			
Crude protein	40.26%	40.13%	40.14%
Crude lipid	9.71%	9.78%	9.72%
Ash	5.37%	5.48%	5.45%
Moisture	6.48%	6.72%	6.52%
Se content	0.05 mg/Kg	0.43 mg/kg	0.82 mg/kg

*^1^Casein purchased from Sigma (St. Louis, MO, United States). ^2^Fish oil purchased from Tongwei Feed Co. Ltd. (Wuxi, China). ^3^Lecithin oil purchased from Zouping Xinglong BIO-Tech Co. Ltd. (Shandong, China). ^4^Vitamin premix (mg/kg dry diet): V_*A*_ 10, V_*D*_ 0.05, V_*E*_ 400, V_*K*_ 40, V_*B1*_ 50, V_*B2*_ 200, V_*B3*_ 500, V_*B6*_ 50, V_*B7*_ 5, V_*B11*_ 15, V_*B12*_ 0.1, V_*C*_ 1,000, inositol 2000, choline 5,000. ^5^Mineral premix (mg/kg dry diet): FeSO_4_ ⋅ 7H_2_O 372, CuSO_4_ ⋅ 5H_2_O 25, ZnSO_4_ ⋅ 7H_2_O 120, MnSO_4_ ⋅ H_2_O 5, MgSO_4_ 2475, NaCl 1875, KH_2_PO_4_ 1000, Ca (H_2_ PO_4_)_2_ 2500.*

### Experimental Design and Sampling

The crabs were farmed in a monoculture. Juvenile crabs weighing 2.41 ± 0.25 g were randomly divided into three groups and placed in plastic boxes (34 cm × 22 cm × 18 cm). Each crab was placed in a separate plastic box and acclimated for 10 days. The crabs were fed commercial feed during acclimation. We selected crabs with a similar size and complete appendages for the formal experiment. Each experimental group consisted of 80 crabs in 80 independent rearing plastic boxes. During the experiment, each plastic box was cleaned once a day at 15:10. The crabs were supplied with experimental feed twice a day (at 9:00 and 17:00) at 5% of body weight, and the residual feed after 2 h was collected, dried, and weighed. One-third of the water was changed every 2 days. During the rearing period, the dissolved oxygen was maintained above 5.5 mg/L, the water temperature was 26.8–29.0°C, the ammonia nitrogen was <0.01 mg/L, and the pH was 7.5 ± 0.2. The crabs were farmed under a natural light cycle, and the whole experimental period was 60 days. At the end of the rearing experiment, feeding was stopped for 1 day, and each crab was weighed separately. We removed the carapace, cut the internal bones and feet from the base of the breastplate, and removed the hepatopancreas and abdominal muscles from 20 crabs per treatment group. These were quick-frozen in liquid nitrogen and then stored at −80°C until analysis.

After the rearing experiment, to reduce the sampling stress, the juvenile crabs were kept in plastic boxes for 10 days. Cu^2+^ was then added to the water in the form of CuSO_4_.5H_2_O (analytical product). A stock solution was prepared with double-distilled water and then diluted to the experimental concentration. Based on the results of preliminary experiments, the Cu^2+^ concentration at which death occurred by 96 h (0.24 mg/L) was selected as the exposure concentration for this study. The Cu^2+^ concentration in water was measured by ICP-MS. Juvenile crabs in the different treatment groups were exposed to 0.24 ± 0.02 mg/L Cu^2+^ for 96 h. During the experiment, the Cu^2+^ concentrations in the water were adjusted every 12 h. At 12, 24, 48, and 96 h of Cu^2+^ stress, nine crabs in each experimental group were randomly selected, and their hepatopancreases were removed and washed with ice-cold saline. The samples were divided into two portions, which were immediately frozen in liquid nitrogen and stored at −80°C until analyses of physiological and biochemical indexes and gene expression had been performed.

### Analysis of Measurement Parameters

#### Analysis of Growth Parameters

The calculations for the growth parameters were:

Specificgrowthrate(SGR,%):SGR=(InFBW-InIBW)/T*100,

where FBW = final body weight (g); IBW = initial body weight (g); and *T* is the number of test days.

Feed⁢conversion⁢ratio⁢(FCR)

=feed⁢intake⁢(g/crab)/wet⁢weight⁢gained⁢(g)

Survival(S,%)=(no.ofcrabharvested/no.ofcrabstocked)

*100

Hepatosomaticindex(HSI,%)

=(hepatopancreas⁢weight/whole⁢body⁢weight)*100

#### Analysis of Hepatopancreas Enzyme Activity and Biochemical Parameters

The hepatopancreas samples were selected from each experimental group. Each sample (0.6 g) was homogenized for 30 s with 3 mL (W:V = 1:5) pre-chilled normal saline using a micro-homogenizer (T10B, IKA, Staufen, Germany). The homogenate was centrifuged for 20 min at 4°C at 12,000 r/min. The supernatant was collected and centrifuged again, and the resulting supernatant was used for further analyses. Kits purchased from the Nanjing Jiancheng Biotechnology Research Institute (Nanjing, China) were used to measure the activities of trypsin, pepsin, amylase, and lipase. The activity of tryptase and pepsin is defined in the following way: when the pH is 8.0 at 37°C, the enzyme contained in 1 mg of protein changes the absorbance by 0.003 per min to 1 unit of enzyme activity (U). Amylase activity is defined in the following way: at 37°C, 1 mg of protein interacts with the substrate for 30 min, and hydrolyzes 10 mg of starch as 1 unit of enzyme activity (U). Lipase activity is defined in the following way: at 37°C, 1 g of tissue protein reacts with the substrate for 1 min and consumes 1 μmol of substrate.

We determined SOD, CAT, GSH-Px, GR, and malondialdehyde (MDA) in the hepatopancreas according to Wang et al. (2020). Peroxidase activity (POD) in the hepatopancreas was measured according to Espinosa et al. (2020). The determination of ACP is based on the fact that ACP can decompose disodium phosphate into free phenol and phosphoric acid. Phenol can be reacted with 4-aminoantipyrine in alkaline solution. Finally, it is oxidized by potassium ferricyanide to produce a red quinone derivative, and then the activity of ACP enzyme is measured. The alkaline phosphatase (ALP) activities of hepatopancreas were detected using a phosphatase assay kit operated in accordance with procedures outlined by Liang et al. (2020). γ-glutamyl transpeptidase (γ-GT) was determined according to the procedure described by Chen et al. (2012), and the nitric oxide (NO) contents were measured according to procedures in Anderson (1964). All the kits were purchased from Nanjing Jiancheng Biotechnology Research Institute. The Se contents in the hepatopancreas and abdominal muscle samples were measured by ICP-MS.

Another samples (0.1 g) from each experimental group were selected for gene expression analysis. The remaining hepatopancreas samples were used to measure total lipid content according to the method in Folch et al. (1957). The lipid composition was analyzed by thin-layer chromatography (IATROSCAN MK-6s; Iatron Inc., Tokyo, Japan) (Wu et al., 2014).

#### Analysis of Immune-Related Gene Expression

We analyzed expression of immune-related genes including metallothionein (MT), LYZ and heat shock 70 (HSP70) in this study. Total RNA was extracted from the hepatopancreas by the Trizol method. The quality of total RNA was determined by 1% agarose gel electrophoresis, and its concentration and OD260/OD280 values were measured using a Nanodrop ND-2000 spectrophotometer (Thermo Fisher Scientific, Waltham, MA, United States). The OD260/OD280 values ranged from 1.8 to 2.0. A sample of total RNA with clear bands and no obvious drag was used as the template. cDNA was prepared using the Prime Script^®^ RT reagent kit (TaKaRa, Dalian, China). Real-time PCR was performed using an SYBR^®^ Premix Ex Taq^TM^ II kit (TaKaRa). The reaction mixture was 20 μL, and each reaction was performed in triplicate. The thermal cycling conditions were: 95°C, 30 s, and 95°C, 5 s; and 57°C, 30 s, and 72°C, 1 min for 40 cycles, followed by a dissolution reaction at 95°C for 15 s, 60°C for 15 s, and 95°C for 15 s. Each reaction had three replicates. All test samples contained an NC without template to rule out false-positive results. β-actin was used as the reference gene. Relative mRNA levels in mitten crab were calculated using the 2^–ΔΔ*Ct*^ method. Gene primers were designed by Primer Premier 6.0 (MT and HSP70 genes) or related references (LYZ and β-actin genes) (Sun et al., 2012; Qi et al., 2019), and synthesized by Jinweizhi Biotechnology Co., Ltd. (Suzhou, China) (Table 2).

**TABLE 2:** Primer sequences of qRT-PCR.

**Name**	**Primer sequence (5′–3′)**	**Registration number**
Metallothionein	F: 5′- AAGTGCAGCAACAAGGAGGA -3′	GU479377.1
	R: 3′- TGACGCGGTGGTTATCTGTC-5′	
Lysozyme	F: 5′- ATGATGCGTGTGATCTGCC-3′	JN416111.1
	R: 5′- CATGACGCATCCATCGCTTG-3′	
Heat shock 70	F: 5′-ACGTCTTCCACTCGGCATTT-3′	KC493626.1
	R: 5′-GTTCTTGAACACGCCCACAC-3′	
β-actin	F: 5′-GCATCCACGAGACCACTTACA-3′	HM053699.1
	R: 5′-CTCCTGCTTGCTGATCCACATC-3′	

### Data Analysis

The results are expressed as mean ± standard deviation (mean ± SD). Experimental data were subjected to analysis of variance using SPSS 21.0 (SPSS Inc., Chicago, IL, United States). The data were tested for a normal distribution and homogeneity of variance. Paired-samples *t*-test was used to compare different treatment times within the same experimental group, and Duncan’s multiple comparison was used to compare different treatment groups at the same time. The significance level was *P* < 0.05.

## Results

### Effects of Dietary Supplementation With Se-Cultured *P. maackianus* Powder on Growth Performance and Se Content in Hepatopancreas and Muscle of Mitten Crab

The actual Se contents in experimental diets as measured by ICP-MS were 0.05, 0.43, and 0.82 mg/kg (Table 3). After 60 days of rearing, there were significantly higher final weights in both the 0.43 and 0.82 mg/kg groups than in the 0.05 mg/kg group (Table 3). We found higher SGR and lower FCR in the 0.43 mg/kg group, compared with the 0.05 mg/kg group, but SGR and FCR did not differ significantly among the three groups (*P* > 0.05). The survival of juvenile crabs was not significantly different among the three experimental groups (91.5–92.9%; *P* > 0.05). In addition, there was no significant difference in the HSI among the experimental groups. When the Se content in the diet increased from 0.05 to 0.82 mg/kg, the Se content in the hepatopancreas of juvenile crabs increased from 0.42 to 1.87 mg/kg. Similar trends in concentrations of Se were found in muscle (0.21, 0.42, and 0.55 mg/kg in the 0.05, 0.43, and 0.82 mg/kg group, respectively).

**TABLE 3:** Growth and Se content in hepatopancreas and muscle of Chinese mitten crab (*Eriocheir sinensis*) fed dietary supplementation with cultured *Potamogeton maackianus* powder.

	**0.05 mg/L group**	**0.43 mg/L group**	**0.82 mg/L group**
***n* = 20 replicates per group**			
Initial weight (g)	2.36 ± 0.17	2.42 ± 0.13	2.41 ± 0.14
Final weight (g)	8.21^b^ ± 0.75	9.91^a^ ± 0.85	9.82^a^ ± 0.81
Specific growth rate (%/d)	2.08 ± 0.17	2.35 ± 0.18	2.34 ± 0.18
Feed conversion ratio	2.34 ± 0.23	2.06 ± 0.21	2.08 ± 0.19
Hepatopancreas somatic index (%)	11.84 ± 1.52	11.65 ± 1.61	11.14 ± 1.65
Survival (%)	92.5	91.2	93.8
Se contents in hepatopancreas (mg/kg)	0.42^c^ ± 0.01	1.61^b^ ± 0.01	1.87^a^ ± 0.02
Se contents in muscle (mg/kg)	0.21^c^ ± 0.00	0.42^b^ ± 0.01	0.55^a^ ± 0.01

*Data are expressed as mean ± SD (except survival). Means in the same row with different superscripts are significantly different (P < 0.05).*

### Effects of Dietary Supplementation With Se-Cultured *P. maackianus* Powder on Biochemical Parameters and Digestive Enzyme Activity in Hepatopancreas of Mitten Crab

The activities of amylase and trypsin in the hepatopancreas were not significantly different between the 0.82 mg/kg group and the 0.05 mg/kg group, but the activities of pepsin and lipase were significantly higher in the 0.82 mg/kg group than in the 0.05 mg/kg group (Table 4). Lipase activity was also significantly higher in the 0.43 mg/kg group than in the 0.05 mg/kg group; but pepsin and trypsin activities did not differ significantly between these two groups. There was no significant difference in ALP and γ-GT activities between the 0.05 mg/kg group and the 0.43 mg/kg group; but their activities were significantly lower in those two groups than in the 0.82 mg/kg group (*P* < 0.05) (Table 5). As the Se content in the diet rose, the ACP activity and NO content in the hepatopancreas of Chinese mitten crab increased gradually. The ACP activity and NO content in the hepatopancreas of Chinese mitten crab differed significantly among the three experimental groups (*P* < 0.05). The hepatopancreas TC and TG contents were significantly higher in the 0.82 mg/kg group than in the 0.43 mg/kg group and the 0.05 mg/kg group. There was no significant difference in TG content between the 0.43 mg/kg group and the 0.05 mg/kg group (*P* > 0.05).

**TABLE 4:** Digestive enzyme activity in hepatopancreas of Chinese mitten crab (*Eriocheir sinensis*) fed dietary supplementation with cultured *Potamogeton maackianus* powder.

	**0.05 mg/L group**	**0.43 mg/L group**	**0.82 mg/L group**
***n* = 10 replicates per group**			
Trypsin activity (U/mg)	601.53 ± 28.91	597.33 ± 22.43	637.16 ± 27.25
Pepsin activity (U/mg)	0.38^b^ ± 0.02	0.41^b^ ± 0.02	0.53^a^ ± 0.03
Lipase activity (U/mg)	2.15^b^ ± 0.04	2.68^a^ ± 0.07	2.73^a^ ± 0.07
Amylase activity (U/mg)	0.46 ± 0.03	0.39 ± 0.04	0.42 ± 0.03

*Data are expressed as mean ± SD. Means in the same row with different superscripts are significantly different (P < 0.05).*

**TABLE 5:** Biochemical parameters in hepatopancreas of Chinese mitten crab (*Eriocheir sinensis*) fed dietary supplementation with cultured *Potamogeton maackianus* powder.

	**0.05 mg/L group**	**0.43 mg/L group**	**0.82 mg/L group**
**n = 10 replicates per group**			
Acid phosphatase (U/gprot)	5.23^c^ ± 0.54	6.41^b^ ± 0.56	9.49^a^ ± 0.92
Alkaline phosphatase (U/gprot)	35.19^b^ ± 3.87	34.17^b^ ± 3.91	46.38^a^ ± 4.21
γ-glutamyl transpeptidase (U/gprot)	153.14^b^ ± 7.92	169.33^b^ ± 8.11	222.47^a^ ± 15.29
Nitric oxide (umol/gprot)	1.65^b^ ± 0.17	1.81^ab^ ± 0.21	2.01^a^ ± 0.15
Triglyceride (%)	69.35^b^ ± 5.21	72.38^b^ ± 4.63	89.31^a^ ± 6.22
Total cholesterol (%)	1.75^c^ ± 0.07	2.34^b^ ± 0.12	3.78^a^ ± 0.15

*Data are expressed as mean ± SD. Means in the same row with different superscripts are significantly different (P < 0.05).*

### Effects of Dietary Supplementation With Se-Cultured *P. maackianus* Powder on Antioxidant Capacity of Mitten Crab and Its Response Under Cu^2+^ Stress

**FIGURE 1:**
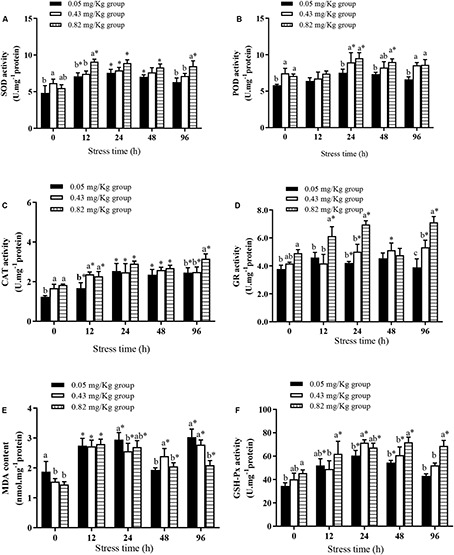
Effects of dietary supplementation with Se-cultured *Potamogeton maackianus* powder on antioxidant capacity of Chinese mitten crab (*Eriocheir sinensis*) and its response under Cu^2+^ stress (*N* ≥ 9). This study is divided into three experimental groups, adding 0.05, 0.43, and 0.82 mg/kg Se to the feed. After 60 days of rearing experiment, hepatopancreas tissues of 10 fish were taken for determination of SOD **(A)**, POD **(B)**, CAT **(C)**, GR **(D)**, MDA **(E)**, and GSH-Px **(F)** levels/activities. At 12, 24, 48, and 96 h of Cu^2+^ stress, nine crabs in each experimental group were randomly selected, and their hepatopancreases were taken for determination of SOD, POD, CAT, GR, MDA, and GSH-Px levels/activities. ^∗^indicates significant differences between values obtained before and after injection (paired-samples *t* test; *P* < 0.05). Different lowercase letters show significant differences among different treatments at each sampling point (Duncan’s multiple range test; *P* < 0.05).

After 60 days of an Se-containing diet, the SOD activity in the hepatopancreas was significantly higher in the 0.43 mg/kg group than in the 0.05 mg/kg group and the 0.82 mg/kg group (Figure 1A). The activity of CAT in the hepatopancreas was significantly lower in the 0.05 mg/kg group than in the 0.43 mg/kg group and the 0.82 mg/kg group (*P* < 0.05) (Figure 1C), but did not differ significantly between the 0.43 mg/kg group and the 0.82 mg/kg group (*P* > 0.05). The MDA content was significantly higher in the 0.05 mg/kg group than in the 0.43 mg/kg group and the 0.82 mg/kg group (*P* < 0.05) (Figure 1E), and the POD (Figure 1B), GR (Figure 1D), and GSH-Px (Figure 1F) activities were significantly higher in the 0.82 mg/kg group than in the 0.05 mg/kg group (*P* < 0.05).

At the end of the rearing experiment, the crabs were subjected to acute Cu^2+^ stress conditions for 96 h. Three crabs in the 0.05 mg/kg group died, but none died in the other experimental groups. The activities of SOD and CAT in each experimental group tended to increase in the first 24 h of Cu^2+^ stress. From 24 to 48 h, SOD and CAT activities in each experimental group were relatively stable, but were significantly higher than their pre-stress activities. In all groups, the MDA content in the hepatopancreas first increased, then decreased, then increased by 96 h of Cu^2+^ stress. All groups showed a sharp increase in MDA content in the first 12–24 h of Cu^2+^ stress. At 48 h, the MDA content was not significantly different from the pre-stress level in the 0.05 mg/kg group, but was significantly higher than pre-stress levels in the 0.43 mg/kg group and the 0.82 mg/kg group (*P* < 0.05). At 96 h of Cu^2+^ stress, the MDA content in the hepatopancreas was significantly lower in the 0.82 mg/kg group than in the 0.05 mg/kg group and the 0.43 mg/kg group. At 12 h of Cu^2+^ stress, the POD activities in all experimental groups were not significantly different from pre-stress activities, and POD activity was significantly higher in the 0.82 mg/kg group than in the 0.05 mg/kg group at 24, 48, and 96 h of Cu^2+^ stress. There was no significant difference in POD activity in the 0.05 mg/kg group during the 96-h Cu^2+^ stress period (*P* > 0.05). At 12 h and 24 h of Cu^2+^ exposure, GR activity in the 0.82 mg/kg group was significantly higher than pre-stress activity, and significantly higher in the 0.82 mg/kg group than in the other experimental groups. At 48 h of Cu^2+^ exposure, there was no significant difference in GR activity among the three experimental groups (*P* > 0.05). At 48 h and 96 h of Cu^2+^ stress, the GSH-Px activities were significantly higher in the 0.82 mg/kg group than in the 0.05 mg/kg group and the 0.43 mg/kg group (*P* < 0.05).

### Effects of Dietary Supplementation With Se-Cultured *P. maackianus* Powder on Expression of Genes Encoding *MT*, *LYZ*, and *HSP70* in Mitten Crab Under Cu^2+^ Stress

**FIGURE 2:**
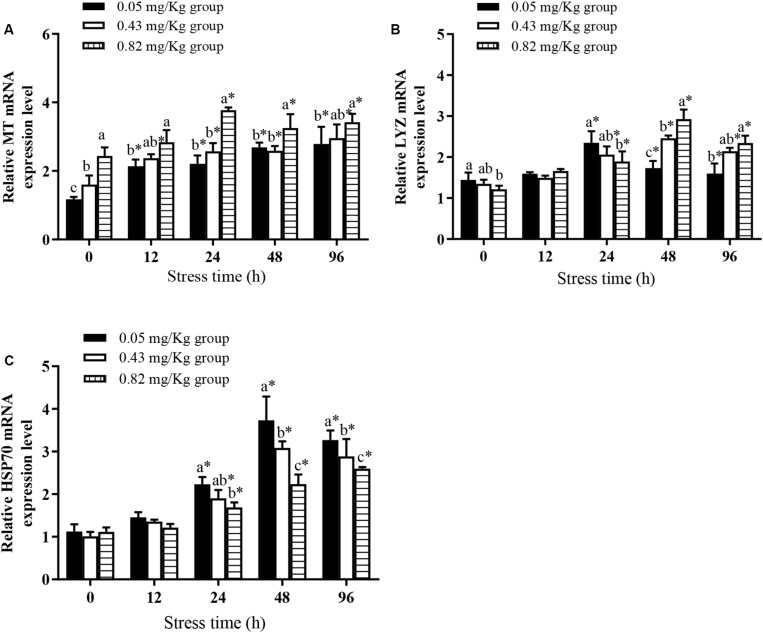
Effects of dietary supplementation with Se-cultured *P. maackianus* powder on *MT* **(A)**, *LYZ* **(B)**, and *HSP70* **(C)** gene expression of Chinese mitten crab (*E. sinensis*) and its response under Cu^2+^ stress (*N* ≥ 9). This study is divided into 3 experimental groups, adding 0.05, 0.43, and 0.82 mg/kg Se to the feed. After 60 days of rearing experiment, hepatopancreas tissues of 10 fish were taken for determination of *MT*, *LYZ*, and *HSP70* gene expression. At 12, 24, 48, and 96 h of Cu^2+^ stress, nine crabs in each experimental group were randomly selected, and their hepatopancreases were taken for determination of *MT*, *LYZ*, and *HSP70* gene expression. ^∗^indicates significant differences between values obtained before and after injection (paired-samples *t* test; *P* < 0.05). Different lowercase letters show significant differences among different treatments at each sampling point (Duncan’s multiple range test; *P* < 0.05).

At the end of the rearing experiment the transcript level of *MT*, encoding metallothionein (Figure 2A), was significantly higher in the 0.82 mg/kg group than in the other groups. The transcript levels of *LYZ* (Figure 2B) and *HSP70* (Figure 2C) were not significantly different between the 0.43 mg/kg group and the 0.05 mg/kg group, but the transcript level of *LYZ* was significantly higher in the 0.05 mg/kg group than in the 0.82 mg/kg group (*P* < 0.05).

Under Cu^2+^ stress, *MT* transcript levels in mitten crabs in each experimental group increased significantly. Within 12–96 h of Cu^2+^ stress, the *MT* transcript levels in each treatment group were significantly higher than the pre-stress levels. The transcript level of *MT* was significantly higher in the 0.82 mg/kg group than in the 0.05 mg/kg group at all sampling times (*P* < 0.05). The transcript levels of *LYZ* in each experimental group increased first and then decreased, with peak levels at 24 h in the 0.05 mg/kg group and at 48 h in the 0.43 mg/kg group and the 0.82 mg/kg group. The *LYZ* transcript levels were significantly higher in the 0.82 mg/kg group than in the 0.05 mg/kg group at 12, 48, and 96 h of Cu^2+^ stress (*P* < 0.05). The transcript levels of *HSP70* continuously increased in the 0.82 mg/kg group during the 96 h of Cu^2+^ stress; those in the 0.05 mg/kg group and the 0.43 mg/kg group increased to peak at 48 h of Cu^2+^ stress and then decreased. In addition, the *HSP70* transcript levels were significantly higher in the 0.05 mg/kg group than in the 0.82 mg/kg group at 48 and 96 h (*P* < 0.05).

## Discussion

### Increasing Se Content Promoted Growth Performance and Se Accumulation

The growth performance of Chinese mitten crab significantly increased when the diets were supplemented with 0.43 mg/kg Se. The positive growth responses to Se supplementation of this result are consistent with those reported for Tian et al. (2014); Qin et al. (2016), and Kong et al. (2017). Se mainly improves animal growth performance because (1) Se is an important component of GSH-Px, which can reduce the body’s stress response, improve antioxidant capacity, enhance the body’s immune function, reduce mortality, and then promote growth (Tian et al., 2014); and (2) Se is a component of the enzyme 5′-deiodinase. An appropriate amount of Se in the diet can activate the synthesis and metabolism of thyroxine and catalyze the conversion of T4 to T3, which controls the gene expression and synthesis of growth hormone, and is beneficial for animal growth (Ibrahim et al., 2011). Thus, the improvement in growth performance and feed utilization in the 0.43 mg/kg Se-supplemented groups might be associated with the function of Se.

However, our study indicated that, as the Se content in the diet increased, the weight of Chinese mitten crab did not continue to increase. Adding Se to the diet may have both nutritional and toxic effects. Excess dietary Se may cause Se deficiency symptoms or slow or acute toxicity in aquatic animals. Kong et al. (2017) found that excess Se may generate free radicals and cause oxidative damage, thereby affecting normal growth and development in oriental river prawn. However, Wang et al. (2019) found that excess Se-yeast in the diet of Chinese mitten crab reduced oxidative stress in the hepatopancreas, and did not limit growth. Similarly, the organic Se we added to the diets came from plants, which may have fewer toxic effects on mitten crab. Yuan et al. (2018) also found that Se-biofortified corn fed to mitten crab was more readily biologically active while being less toxic.

The Se content in the hepatopancreas and muscle of juvenile crabs increased with rising levels of Se in the diet, indicating that the crab body was able to become enriched with Se from the diet. Organic Se can increase the body’s total Se accumulation (Yuan et al., 2018). Differences in metabolic pathways between organic Se and inorganic Se can affect Se accumulation (Kouba et al., 2014). Organic Se is similar to sulfur-containing amino acids. In protein synthesis, seleno-amino acids often replace sulfur-containing amino acids and are incorporated into proteins, which can increase the Se storage capacity (Schram et al., 2008). Therefore, adding Se-enriched plants to increase the content of organic Se in diets can effectively promote the ready accumulation of Se, which also provides a new strategy for the addition of organic Se in future.

### Increasing Se Content Regulated the Activities of Digestive Enzymes and Immune Enzymes

In this study, pepsin and lipase activities were significantly lower in the 0.05 mg/kg group than in the 0.82 mg/kg group. Similar results have been found in studies on aquatic animals. For example, dietary supplementation with 0.6% Se-yeast increased protease activity in the hepatopancreas of Gibel carp (Liu et al., 2002). When 0.6 mg/kg Se was added to the diet, the activities of protease, amylase, and lipase were significantly increased in the hepatopancreas of grass carp (*Ctenopharyngodon idellus*) (Su et al., 2007). Proteases and lipases produced by the hepatopancreas determine digestive ability, which directly affects the digestion and absorption of nutrients such as proteins and fats (Qiang et al., 2017). In mammals, Se deficiency can impair the hepatic and pancreatic acinar and islet function and reduce acinar secretion, thereby inhibiting amylase and lipase activity (Zhang et al., 1997). The organic Se content in the diets of the 0.43 mg/kg group and the 0.82 mg/kg group was considered to facilitate adequate digestibility and consequent enzyme activity in the Chinese mitten crab hepatopancreas, enhance fat intake and metabolic capacity, and increase the accumulation of TG and TC.

The two important phosphatases ACP and ALP have important effects on the body’s metabolism and immunity. In mitten crab, feeding with 0.5 mg/kg Se-yeast stimulated ACP activity, suggesting that the immune system was activated *in vivo* (Wang et al., 2019). In addition, mitten crab fed on a fermented diet showed increased ACP phosphatase activity, and increased total protein and globulin contents in the hemolymph (Xu et al., 2019). In this study, we detected higher ACP activity in the hepatopancreas in the 0.43 mg/kg group, and this may have been related to the activated immune capacity of juvenile crabs. However, as levels of Se in feed increased (0.82 mg/kg), ACP activity was not inhibited, and this differed from the results of Wang et al. (2019). Different Se sources and differences in farming environments may affect crab immune response regulation. The addition of 0.82 mg/kg Se feed did not exceed the upper limit of addition, so there was no inhibitory effect. The biologically active compound NO can act as an antioxidant and anti-apoptotic substance to inhibit programmed cell death (Beligni and Lamattina, 1999). In our study, the inclusion of 0.05 mg/kg Se in the diet led to significantly inhibited activities of ACP, ALP, and γ-GT in juvenile crabs, and suppressed their immune function. The lower NO content in the body of 0.05 mg/kg group may be related to inhibition of the antioxidant system.

### Increasing Se Content Promoted Antioxidant Regulation Before and After Stress

Our study found that the MDA content in juvenile crab was significantly lower in the 0.82 mg/kg group than in the 0.43 mg/kg group. The product of lipid peroxidation, MDA, is often used as an important indicator of oxidative damage. Activation of GSH-Px activity by Se has been shown to prevent lipid peroxidative damage in the hepatopancreas (Kouba et al., 2014). Similar results were found in Yuan et al. (2018) and Kong et al. (2017). The deposition of Se in the body acts as a reservoir for rapid utilization under oxidative stress, and promotes the biological activity of antioxidant enzymes (Kim and Kang, 2014).

Chinese mitten crabs inhabit the bottom of ponds, where contamination by heavy metals is more likely to occur. Therefore, at the end of the rearing experiment, the mitten crabs in the three treatment groups were subjected to Cu^2+^ stress for 96 h. The activities of GSH-Px and SOD, and their balance, play an important role in resistance to oxidative damage in the body. Together with CAT, they are the first line of cellular defense (Liu et al., 2018). The higher SOD, POD, GR, and GSH-Px activities in the 0.82 mg/kg group than in the other groups may have been more effective in removing free radicals under Cu^2+^ stress, leading to lower MDA contents and protection of the cell membrane. Our results show that oxidative stress caused by high Cu^2+^ can be alleviated by feeding Chinese mitten crab with a diet containing increased levels of Se. Wang et al. (2019) also found that the addition of 1 mg/kg Se-yeast in the diet increased activities of SOD and GSH-Px in Chinese mitten crab, reduced lipid peroxides, and enhanced resistance against nitrite stress compared with a 0.5 mg/kg Se-yeast group. Dose correction can balance Se and Cu^2+^ in animal bodies. Cu^2+^ is the structural center of SOD, and uptake of exogenous Cu^2+^ may activate SOD activity (Qiang et al., 2019). Therefore, at 48 h of Cu^2+^ stress, increased SOD and CAT activities in the 0.05 mg/kg group may reduce MDA accumulation in the hepatopancreas tissue of Chinese mitten crab. However, as the stress time increases, the SOD, CAT, GSH-Px, and GR activities of the 0.05 mg/kg group were significantly inhibited, and the MDA content was increased. Lack of Se in the body may result in limited resistance to the free radicals caused by Cu^2+^ stress, thereby leading to increased oxidative stress.

### Increasing Se Content Regulated Gene Expression Before and After Stress

Metallothionein is a low-molecular-weight, cysteine-rich protein that is ubiquitous in organisms. It has a highly conserved structure, and plays an important role in maintaining homeostasis and detoxification (Felix-Portillo et al., 2014). In this study, juvenile crabs fed a diet containing 0.82 mg/kg Se showed a more rapid and continued response of *MT* expression under Cu^2+^ stress. The oxidative stress induced by Cu^2+^ exposure may be alleviated by combining active oxygen radicals with reduced sulfhydryl-enriched MT. In addition, a significant feature of MT is that it contains many cysteine residues, so Se enrichment of the hepatopancreas may help to stimulate *MT* expression (Hao et al., 2019). Supplementation with Se (2–4 mg/kg) can promote *MT* gene expression in the liver of turbot (*Scophthalmus maximus*) fed a diet rich in copper (1,000 mg/kg), leading to the relief of oxidative of stress and the maintenance of homeostasis (Hao et al., 2019). However, further research is required to explore the mechanism by which Se induces MT, and to determine whether it preferentially induces the synthesis of metal selenoproteins or demetallic MTs with different characteristics.

The activity of LYZ is directly related to the immunity and health of aquatic animals. In the present study, the transcript level of *LYZ* in the hepatopancreas was 17% lower in the 0.82 mg/kg group than in the 0.05 mg/kg group at the end of the rearing experiment. LYZ exists in lysosomes before it is secreted into body fluids. Se reduces LYZ activity in aquatic animals in the absence of stimulation by an antigen. This has been attributed to the ability of Se to protect the structural integrity of lysosomes, thereby reducing the amount of LYZ secreted (Low and Sin, 1999). However, when crabs were stimulated by exogenous Cu^2+^, Se facilitated the release of LYZ, suggesting that it can directly improve the activity of non-specific immune factors and enhance resistance to stress. The increased LZM activity of serum in mitten crab fed with increased levels of Se can improve immune response under nitrite stress (Wang et al., 2019). Alina et al. (2009) also found that Se in the diet can be assimilated into enzymes and proteins, which help to improve immunity. Interestingly, we found that the transcript level of *LYZ* in juvenile crabs increased sharply in the 0.05 mg/kg group within 24 h of Cu^2+^ stress, suggesting that Cu^2+^ caused severe oxidative stress in this group. The accumulation of lipid peroxides destroys cell membranes and promotes the release of a large amount of LYZ to relieve oxidative damage.

HSP70 plays an important role in protecting cells from damage and/or restores function to damaged cells, and can be used as an indicator of the physiological state of crustaceans (Han et al., 2018). At the end of rearing experiment, the transcript levels of *HSP70* were significantly higher in the 0.05 mg/kg group than in the other groups, indicating that a lack of Se may regulate the expression of *HSP70*. This is consistent with the findings in turbot (Hao et al., 2019). After adding different levels of Se to the feed containing 1,000 mg/kg Cu^2+^, the expression of hepatic *HSP70* in the 0 mg/kg Se group was found to be significantly higher than that of the groups with 2 and 4 mg/kg Se (Hao et al., 2019), suggesting that the addition of Se in the diet can regulate the expression of *HSP70* gene to protect liver tissue. However, higher Se content in hepatopancreas tissue in juvenile crabs led to rapid up-regulation of *HSP70* under Cu^2+^ stress; higher expression of *HSP70* may help to relieve oxidative stress damage.

## Conclusion

The blade of *P. maackianus* is large and brittle, rich in various nutrients, and is a food source for mitten crabs. Adding Se-containing *P. maackianus* powder to the diet can improve its nutritional status, while also supplementing the diet with Se. Increasing Se content in the diet can promote the growth of mitten crab, reduce the transcript levels of *HSP70* and *LYZ* genes, and induce a rapid and durable response of antioxidant enzymes and *MT* expression under Cu^2+^ stress. Our results show that a high Se content in the hepatopancreas of juvenile crabs leads to increased GSH-Px and GR activities in the antioxidant system, and activation of *MT* transcription. However, further research is required to determine the mechanism by which Se regulates *MT* transcription.

## Data Availability Statement

All datasets generated for this study are included in the article/Supplementary Material.

## Ethics Statement

The study protocols and design were approved by the Ethics Committee at the Freshwater Fisheries Research Centre of the Chinese Academy of Fishery Sciences (Wuxi, China). The juvenile crabs were maintained in well-aerated water and treated with 200 mg/L tricaine methanesulfonate (Sigma, St. Louis, MO, United States) for rapid deep anesthesia. The samples were extracted based on the Guide for the Care and Use of Laboratory Animals in China.

## Author Contributions

PX and JQ conceived and designed the experiments. JH and X-JD cultured *P. maackianus* and fed juvenile crabs. Y-FT and J-WB devised the experimental diets. J-WB, JH, and Y-FT collected samples and measured biochemical parameters. J-WB conducted qRT-PCR experiments. H-JZ and C-KZ measured Se content. JQ and H-JZ analyzed the data. JQ and C-KZ wrote the manuscript with contributions from all other authors. All authors read and approved the final version of the manuscript.

## Conflict of Interest

The authors declare that the research was conducted in the absence of any commercial or financial relationships that could be construed as a potential conflict of interest.
