# Supplementary material for: Selenium-Cultured Potamogeton maackianus in the Diet Can Alleviate Oxidative Stress and Immune Suppression in Chinese Mitten Crab (Eriocheir sinensis) Under Copper Exposure
Source: Front Physiol. 2020 Jun 19;11:713. doi: 10.3389/fphys.2020.00713 (PMC7325926; doi:10.3389/fphys.2020.00713)
Supplement: Supplementary file 1 [file Data_Sheet_1.DOCX]

| Groups | Proximate composition of *Potamogeton maackianus* (% wet weight) | | | | | |
| --- | --- | --- | --- | --- | --- | --- |
|  | Moisture /% | Crude protein /% | Crude fat /% | Crude fiber /% | Ash /% | Total sugar /% |
| 0.02 mg/kg | 82.37±0.17 | 3.48±0.18 | 0.71±0.09 | 2.39±0.02 | 2.43±0.03 | 8.54±0.37 |
| 8.83 mg/kg | 81.81±0.23 | 3.39±0.15 | 0.73±0.08 | 2.37±0.02 | 2.49±0.04 | 8.82±0.32 |
| 16.92 mg/kg | 82.79±0.31 | 3.28±0.18 | 0.76±0.09 | 2.29±0.02 | 2.41±0.03 | 8.55±0.37 |

Table S1 Comparison of proximate composition of three type culture for

*Potamogeton maackianus* (% wet weight)

Table S2 Comparison of fatty acid composition of three type culture for

*Potamogeton maackianus* (% total fatty acids)

| Fatty acid composition | 0.02 mg/kg | 8.83 mg/kg | 16.92 mg/kg |
| --- | --- | --- | --- |
| C14:0 | 0.97±0.01 | 0.92±0.01 | 0.91±0.02 |
| C15:0 | 0.41±0.03 | 0.40±0.03 | 0.47±0.02 |
| C16:0 | 22.37±1.28 | 21.55±1.02 | 22.16±1.15 |
| C17:0 | 0.24±0.02 | 0.22±0.01 | 0.22±0.02 |
| C18:0 | 3.51±0.05 | 3.28±0.02 | 3.47±0.03 |
| C20:0 | 0.82±0.03 | 0.84±0.02 | 0.88±0.02 |
| C22:0 | 0.85±0.02 | 0.88±0.02 | 0.86±0.03 |
| C16:1 | 2.53±0.08 | 2.69±0.05 | 2.71±0.06 |
| C17:1 | 0.33±0.02 | 0.32±0.02 | 0.38±0.01 |
| C18:1n9 | 5.38±0.16 | 5.49±0.24 | 5.15±0.17 |
| C20:1 | 0.07±0.01 | 0.08±0.01 | 0.07±0.01 |
| C22:1n9 | 0.42±0.03 | 0.39±0.04 | 0.41±0.02 |
| C18:2n6 | 22.37±0.18 | 23.01±0.22 | 22.85±0.24 |
| C18:3n6 | 0.45±0.02 | 0.49±0.03 | 0.43±0.02 |
| C18:3n3 | 22.38±0.22 | 22.12±0.14 | 21.59±0.16 |
| C20:2 | 0.12±0.01 | 0.11±0.01 | 0.11±0.01 |
| C20:3n3 | 0.35±0.02 | 0.39±0.01 | 0.37±0.01 |
| C20:4n6 | 0.26±0.01 | 0.24±0.01 | 0.24±0.01 |
| C22:2 | 0.24±0.01 | 0.28±0.01 | 0.25±0.01 |
| C20:5n3 | 1.53±0.04 | 1.51±0.03 | 1.49±0.04 |
